# Validation of a claims-based algorithm identifying eligible study subjects in the ADAPTABLE pragmatic clinical trial

**DOI:** 10.1016/j.conctc.2018.11.001

**Published:** 2018-11-10

**Authors:** Ezra Fishman, John Barron, Jade Dinh, W. Schuyler Jones, Amanda Marshall, Rebecca Merkh, Holly Robertson, Kevin Haynes

**Affiliations:** a HealthCore, Inc., Wilmington, DE, USA; b Duke Clinical Research Institute, Durham, NC, USA

**Keywords:** Cardiovascular disease, Aspirin, Real-world evidence, Acute myocardial infarction, Computable phenotype

## Abstract

**Objective:**

Validate an algorithm that uses administrative claims data to identify eligible study subjects for the ADAPTABLE (Aspirin Dosing: A Patient-centric Trial Assessing Benefits and Long-Term Effectiveness) pragmatic clinical trial (PCT).

**Materials and methods:**

This study used medical records from a random sample of patients identified as eligible for the ADAPTABLE trial. The inclusion criteria for ADAPTABLE were a history of acute myocardial infarction (AMI) or percutaneous coronary intervention (PCI) or coronary artery bypass grafting (CABG), or other coronary artery disease (CAD), plus at least one of several risk-enrichment factors. Exclusion criteria included a history of bleeding disorders or aspirin allergy. Using a claims-based algorithm, based on International Classification of Diseases, 9th Edition, Clinical Modification (ICD-9-CM) and 10th Edition (ICD-10) codes and Current Procedural Terminology (CPT) codes, we identified patients eligible for the PCT. The primary outcome was the positive predictive value (PPV) of the identification algorithm: the proportion of sampled patients whose medical records confirmed their ADAPTABLE study eligibility. Exact 95% confidence limits for binomial random variables were calculated for the PPV estimates.

**Results:**

Of the 185 patients whose medical records were reviewed, 168 (90.8%; 95% Confidence Interval: 85.7%, 94.6%) were confirmed study eligible. This proportion did not differ between patients identified with codes for AMI and patients identified with codes for PCI or CABG.

**Conclusion:**

The estimated PPV was similar to those in claims-based identification of drug safety surveillance events, indicating that administrative claims data can accurately identify study-eligible subjects for pragmatic clinical trials.

## 1 Introduction

Pragmatic clinical trials (PCTs) have the potential to enhance comparative effectiveness research because they can be faster, less costly, and more reflective of the real world than traditional randomized clinical trials, showing the performance of therapies under usual – rather than ideal – conditions [[1], [2], [3]]. However, identification of patients for recruitment into these trials can be challenging. Administrative claims data could serve as a valuable resource to identify patients who may be eligible to participate.

Administrative claims data have been widely used in different types of epidemiologic research [4,5] but their use in PCTs has been limited. Because claims data originate in numerous health systems and healthcare providers, not just those associated with a particular clinical trial, they can offer a broader overview of patients’ utilization histories relative to electronic health records (EHRs) from a single health system. As a result, claims data – though not as rich in clinical detail as EHR data – have the potential to more comprehensively identify patients who may qualify for PCTs than EHR data alone [6].

A key barrier to the incorporation of claims data into PCTs is the lack of validated claims-based algorithms to identify trial-eligible patients. The extent to which the diagnosis and procedure codes appearing in claims data can accurately identify patients eligible for PCTs is unknown, as no study has validated a complete claims-based algorithm identifying patients eligible to be PCT subjects.

The primary objective of this study was to validate a claims-based algorithm for identifying patients eligible for inclusion in the ADAPTABLE (Aspirin Dosing: A Patient-centric Trial Assessing Benefits and Long-Term Effectiveness) clinical trial against the “gold standard” of medical record review. ADAPTABLE is a PCT that aims to identify the optimal dose of aspirin for the prevention of ischemic events in patients with atherosclerotic cardiovascular disease (ASCVD). Aspirin is associated with significant reductions in ischemic outcomes such as AMI and stroke in patients with previous cardiovascular events and/or atherosclerosis [7]; however, despite dozens of clinical trials involving more than 200,000 patients, the optimal dose of aspirin — the most effective dose for reducing ischemic events in secondary prevention, balanced against the potential for adverse events such as gastrointestinal bleeding — has not been determined in direct comparative effectiveness trials [8,9]. Doses of 81 mg/day and 325 mg/day are commonly prescribed after AMI; however, there is no consensus about which dose is optimal for which patients [8,10].

In an attempt to move the field toward such a consensus, ADAPTABLE is randomly assigning patients with heart disease to receive an aspirin dose of 81 mg/day or 325 mg/day. The ADAPTABLE study is conducted under the aegis of the Patient Centered Outcomes Research Network (PCORnet), a coordinated network of health systems that collects and stores clinical data in a common data model (CDM) that standardizes data domains and definitions [11].

The results of this validation study will provide a case study of the extent to which claims data can be used to identify patients eligible for PCTs.

## 2 Materials and methods

### 2.1 Source population

We identified a cohort of eligible ADAPTABLE patients by running a computer algorithm developed by the ADAPTABLE study team on our claims database. The claims data were structured according to the PCORnet common data model (CDM), with a date range of 01/01/2006 to 04/01/2017.

The algorithm, code for which is publicly available at https://github.com/ADAPTABLETRIAL/PHENOTYPE, [12] applies the ADAPTABLE inclusion and exclusion criteria to all patients in our database. Subjects are eligible for the ADAPTABLE study if they had a prior AMI, a prior revascularization procedure (percutaneous coronary intervention [PCI] or coronary artery bypass grafting [CABG]), a prior angiogram showing significant coronary artery disease (CAD), or a history of chronic ischemic heart disease, CAD, or ASCVD. In addition to one of the above, subjects also need at least one of the following characteristics, which are known as enrichment factors: Age at least 65 years, creatinine at least 1.5 mg/dL, prevalent diabetes mellitus, known 3-vessel CAD, cerebrovascular disease, peripheral arterial disease, left ventricular ejection fraction <50%, chronic systolic or diastolic heart failure, systolic blood pressure at least 140 in the past 12 months, low-density lipoprotein at least 130 in the past 12 months, or be a current smoker. Exclusion criteria were a history of significant bleeding, a gastrointestinal (GI) bleeding condition, or an allergy to aspirin (aspirin allergy was not available in claims data). The study inclusion and exclusion criteria have been described elsewhere [13]. Crucially, the study intentionally included *prevalent* cases, that is, individuals who have had cardiac problems for many years, including those who were currently on an aspirin regimen. The study was not limited to individuals experiencing new AMI or cardiac procedures (incident cases), as is typical in safety outcome studies.

Running the algorithm on our claims data generated a list of patients identified as potential ADAPTABLE study subjects. We used this patient list as our starting population.

### 2.2 Case identification

From the starting population, we identified those with prior AMI, prior PCI, and/or prior CABG in facility claims occurring on or after 10/01/2015 (ICD-10 era). The most recent facility claim with a code for AMI, PCI, or CABG was used as the index claim. Although patients with CAD and an enrichment factor are study-eligible, we excluded patients whose claims histories indicated CAD without AMI, PCI, or CABG to enrich our population with more claims-measureable clinical events. We assumed that medical records associated with facility claims would contain more information related to the patient's history with AMI, PCI, and/or CABG than those associated with outpatient providers. We allowed codes for AMI, PCI, or CABG to appear in any position in the claim, so that we could include medical records documenting the history of these study-qualifying events in addition to those documenting new events.

Then we excluded members whose records would not be obtainable due to regulatory limitations. These regulatory exclusions were assumed independent of the potential study outcomes.

For a sufficient sample size to meet the study's secondary objective, we sought to obtain at least 100 medical records from patients identified based on claims for AMI and at least 75 records from patients identified based on claims for PCI or CABG, for a total of at least 175 records. Based on an expected retrieval rate of approximately 60% of requested records [14], we calculated that we would need to request records for 172 patients with AMI and 128 patients with PCI or CABG. From the list of patients remaining after the regulatory exclusions, we randomly selected 172 patients with AMI (regardless of PCI or CABG status) as Stratum 1, and then randomly selected an additional 128 with PCI or CABG (regardless of AMI status) as a Stratum 2. For the selected 300 patients, we prepared a file with identifying information on the patient and the provider from the index claim.

Using the identified patient/provider file, we sent an initial medical record request documentation packet to the facilities associated with the index claims. This request packet included information about the study, documentation of the study's institutional review board (IRB) approval, a “frequently asked questions” sheet, and limited patient information necessary to identify the record being requested. Following the initial request, up to four additional outreaches by fax and/or follow-up telephone call were performed in an attempt to obtain the records. Any record that could not be located after the fifth attempt was classified as unobtainable, as was any record that a facility indicated it could not provide; such records were excluded from the denominator of the positive predictive value calculation [14,15].

Medical records were abstracted using a standardized form (see appendix) by a HIPAA-compliant third-party vendor blinded to study objectives. Abstracted information consisted of: record type (e.g. inpatient discharge note, emergency room discharge note), documentation of each of the cardiac events of interest, and documentation of any of the exclusion criteria (aspirin allergy, GI bleed, or other bleeding disorder). Electronic files of the abstraction data were transferred to HealthCore, where all abstracted data were quality-control checked using HealthCore-generated SAS programs (SAS Institute, Inc., Cary, NC) to find missing values, inconsistencies between abstracted and claims-based patient dates of birth, and other identifiable errors. Electronic copies of the medical records were also obtained.

### 2.3 Case confirmation

As a pilot, the first ten cases were reviewed by the abstractors, HealthCore's Research Data Collection team (JD and RM), and two clinical pharmacists (JB and KH). Admission history and discharge summary data contained sufficient clinical documentation of past medical history of AMI, PCI, or CABG to evaluate ADAPTABLE study eligibility. Upon approval of the data and proven success of the process during the pilot, medical record obtaining and abstraction continued until all 300 requested records were either obtained or determined unobtainable.

After all obtainable records had been abstracted and collected, they were reviewed by the study investigators. Specifically, a random sample of abstraction-confirmed cases and all abstraction-disconfirmed cases were reviewed manually by the two clinical pharmacist experts, who were blinded to each other's reviews of the records. If both experts reported that an abstracted record should be reversed (that is, its status changed from “confirming study eligibility” to “not confirming study eligibility” or vice versa), then the record was reversed. If only one expert reported that an abstracted record should be reversed, then the record was discussed in conference until a consensus was reached.

As a sensitivity analysis, we considered as “confirmed study-eligible” patients whose medical records showed evidence CAD but not evidence of AMI, PCI, or CABG. As explained earlier, patients with CAD and at least one enrichment factor are eligible ADAPTABLE subjects even without a history of AMI, PCI, or CABG, but patients with this pattern in their claims histories were excluded from the validation study.

### 2.4 Statistical analysis

The positive predictive value (PPV) is a proportion where the numerator is the number of patients whose medical record/abstraction indicate study eligibility over a denominator of patients identified by the algorithm whose records were obtained. We calculated the exact (Clopper-Pearson) 95% confidence limits for binomial random variables.

We also estimated a PPV for the patients selected into the AMI stratum and those selected in the PCI/CABG stratum separately. The sensitivity analysis counted in the PPV's numerator patients with CAD but not AMI, PCI, or CABG in their abstracted record.

The ADAPTABLE trial study received Institutional Review Board (IRB) approval from the Duke University Health System IRB for Clinical Investigations (DUHS IRB), and HealthCore obtained IRB approval from the New England Independent Review Board (NEIRB #120170216).

## 3 Results

### 3.1 Sample attrition

As shown in Table 1, approximately 1.4 million people were identified by the algorithm as potentially study-eligible. Of these, 148,072 had at least one facility claim with a code for AMI, PCI, or CABG on or after 10/01/2015. A total of 34,153 people met the regulatory criteria that allowed us to request their medical records. Of those, we sampled 300 people for whom we requested the medical records associated with the index claim.

**Table 1:** Sample size and attrition.

Step	Criterion	Number of subjects (patients)
1	Number of people identified by claims algorithm as study-eligible	1,400,917
2	Of step 1, number of people with prior MI, prior PCI, or prior CABG documented in facility claims	380,312
3	Of step 2, number with claim(s) for services rendered on or after 10/01/2015	148,072
4	Of step 3, number of fully insured members with full contact information for provider linked to index claim^a^	34,153
5	Of step 4, number of people with AMI randomly selected to have information sent to abstractor	172
6	Of those in step 4 who were *not* selected in Step 5, number of people with PCI or CABG randomly selected to have information sent to abstractor	128

a Non-missing identifying patient-level information; sufficient contact information for the facility and provider of record; fully insured members only (administrative-services-only members excluded) because study was not authorized to query the medical records of most administrative-services-only (ASO) members. Patients and providers who requested to be placed on HealthCore "Do-not-call/do-not-contact" lists excluded.

### 3.2 Patient characteristics

**Fig. 1:**
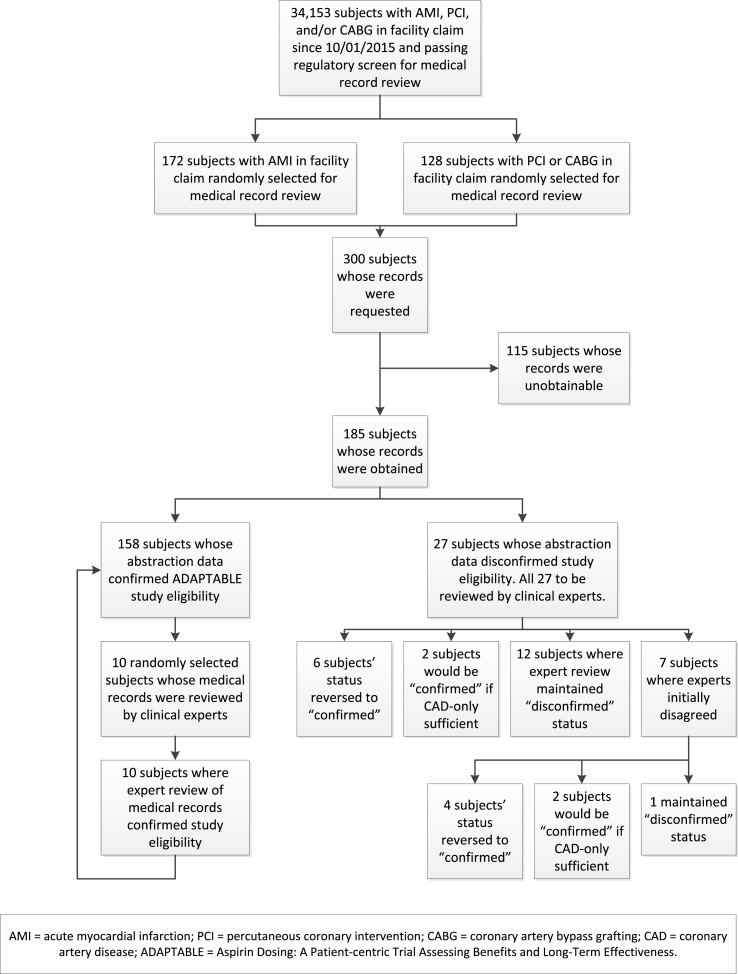
Study process flow.

Of the 300 patients whose medical records were requested, usable records were obtained and abstracted for 185 patients, for a retrieval rate of 61.7%. Characteristics of patients whose records were obtained are shown in Table 2. Characteristics of patients whose records were unobtainable did not differ from those of patients whose records were obtained (Appendix Table 1). A summary of the study process and final status of all 300 requested records is shown in Fig. 1.

**Table 2:** Characteristics of patients whose records were obtained for study-eligibility validation.

Characteristic	N (%) or Mean (SD)
N	185
Age (mean, SD)	74.1 (11.3)
Age>65y (n, %)	150 (81.1)
Female (n, %)	70 (37.8)
Census regions (n, %)	
Northeast	31 (16.8)
South	58 (31.4)
Midwest	60 (32.4)
West	36 (19.5)
Inclusion criteria (n, %):	
History of AMI	146 (78.9)
History of PCI	113 (61.1)
History of CABG	73 (39.5)
History of CAD	176 (95.1)
Enrichment factors (n, %):	
Presence of diabetes	93 (50.3)
Presence of cerebrovascular disease	96 (51.9)
Presence of peripheral artery disease	75 (40.5)
Left ventricular ejection fraction < 50%	39 (21.1)
Creatinine > 1.5 mg/dL	2 (1.1)
systolic or diastolic heart failure	87 (47.0)
LDL cholesterol > 130 mg/dL	8 (4.3)

SD = standard deviation; AMI = acute myocardial infarction; PCI = percutaneous coronary intervention; CABG = coronary artery bypass graft; CAD = coronary artery disease; LDL = low-density lipoprotein.

### 3.3 V**alidation results**

The validation results are shown in Table 3. The abstractors found 158 patient records that confirmed ADAPTABLE study eligibility. This represents a PPV of 0.854 (95% CI: 0.795, 0.902). Our experts (KH and JB) manually reviewed all 27 non-confirmed records plus 10 randomly selected confirmed records.

**Table 3:** Summary of validation results: Positive predictive values, overall and by patient characteristics.

Characteristic	Number of patients identified (denominator)^a^	Number of patients confirmed as ADAPTABLE-eligible (numerator)	Estimated PPV	Exact 95% confidence interval
All patients - abstractor	185	158	0.854	0.795, 0.902
All patients, after clinical expert review (main study result)	185	168	0.908	0.857, 0.946
Sensitivity analysis: Cases of CAD recorded on medical record without AMI, PCI, or CABG counted in the numerator	185	172	0.930	0.883, 0.962
By qualifying events/cardiac history as measured in claims				
Patients in AMI stratum (Stratum 1)	107	97	0.907	0.835, 0.954
Patients in PCI/CABG stratum (Stratum 2)	78	71	0.910	0.824, 0.963
By patient demographic characteristics				
Patients aged < 65 years	35	34	0.971	0.851, 0.999
Patients aged ≥ 65 years	150	134	0.893	0.833, 0.938
Female patients	70	62	0.886	0.787, 0.949
Male patients	115	106	0.922	0.857, 0.964

PPV = positive predictive value; AMI = acute myocardial infarction; PCI = percutaneous coronary intervention; CABG = coronary artery bypass graft; CAD = coronary artery disease.

a Excluding patients whose records were unobtainable.

Of the 27 non-confirmed cases, our two experts independently identified 6 for reversal of status from “non-confirmed” to “confirmed.” For the sensitivity analysis, they identified another 2 that would count as confirmed if patients with CAD only were considered confirmed. The experts also independently verified 12 of the abstractor's non-confirmed cases and all 10 of the abstractor's confirmed cases.

For the remaining 7 of the abstractor's non-confirmed cases, the two clinical experts initially disagreed with each other. Upon conferencing and reading the abstracted records for these 7 patients together, they agreed that 1 was indeed a non-confirmed case, 4 should be reversed to confirmed cases, and 2 would be considered confirmed cases only if CAD-only status counted as a confirmed cases.

The most common reason for our experts’ decision to reverse a record from “non-confirmed” to “confirmed” was the documentation in the medical record that the patient had had an AMI or PCI/CABG in the distant past (e.g. a CABG in 2007). These are *prevalent* cases and thus eligible as study subjects for ADAPTABLE, but were likely not included in the abstraction because the relevant events occurred so far in the past.

Aside from the expert reversals, there were three types of non-confirmed cases: (1) Patients whose medical record documented a condition excluding the patient from ADAPTABLE: two patients with aspirin allergy, plus three patients with GI bleed. (2) Four patients whose medical record documented a history of CAD but not of AMI, CABG, or PCI. (3) Eight patients whose medical records did not have evidence of AMI, CABG, PCI, or CAD; these were often records from non-cardiology encounters such as orthopedic surgeries.

After the experts reviewed the abstractions and the records, the final number of confirmed ADAPTABLE-eligible cases was 168, for a PPV of 0.908 (95% CI: 0.857, 0.946).

As a sensitivity analysis, when cases where a history of CAD found in the medical record was sufficient to count as confirmed, there were 172 confirmed cases and a PPV of 0.930 (0.883, 0.962).

### 3.4 PPV for subgroups

In the AMI stratum, 97 out of 107 patients’ records confirmed their ADAPTABLE-eligibility. In the PCI/CABG stratum, 71 out of 78 cases were confirmed. The ratio of PPVs between the two strata was thus (71/78)/(97/107) = 0.996 (95% CI: 0.908, 1.09). In other words, there was no difference in the PPV between those sampled because of having a code for AMI and those sampled because of having a code for PCI/CABG.

There was also no difference in PPV by sex. For males, 106 out of 115 cases were confirmed. For females, 62 out of 70 cases were confirmed. That produces a male/female ratio of PPVs of 1.041 (95% CI: 0.942, 1.150).

For those aged 65 and older, 134 out of 150 ADAPTABLE-eligible cases were confirmed. For those aged less than 65, 34 out of 35 cases were confirmed. The result is an over-65/under-65 PPV ratio of 0.920 (95% CI: 0.850, 0.996), a marginally significant difference whereby older subjects had a somewhat lower PPV than younger subjects.

## 4 Discussion

Our study identified a 91% PPV for the ADAPTABLE clinical trial patient identification algorithm in the ICD-10 coding period, demonstrating the robustness of claims data in identifying potential subjects for pragmatic clinical trial participation.

The lack of difference in PPV between the groups selected with claims codes for AMI and for PCI/CABG likely arose for two reasons: (1) the overlap in patients’ histories across the two groups: many patients in the AMI stratum had PCI and/or CABG, and many in the PCI/CABG stratum also had AMI; (2) The PCI/CABG stratum included those with *diagnostic* codes for a history of PCI or CABG – not just those with procedure codes. A stratum limited to those with procedure codes for PCI/CABG might have had a higher PPV because the procedure codes refer to the actual procedures, whereas the diagnosis codes reflect providers' knowledge of procedures patients had in the past.

The studies that are most similar to our investigation are Cutrona et al., [4] Cutrona et al., [16] and Ammann et al., [15] which validated AMI codes for drug-safety surveillance. Their estimated PPVs ranged from 75% to 93%, which puts our estimates at the high end relative to the available literature. Our study adds to the literature in several ways. First, we focused on ICD-10 coding, thus updating the validated AMI codes to the present coding system. Furthermore, we included PCI, CABG, and “old” AMI, which were sufficient to qualify a patient for the ADAPTABLE trial but which would have been out of the scope for the other studies. We also identified exclusion criteria and events (GI bleed, aspirin allergy) as reasons for reduced PPV. Individuals with these criteria were identified as study-eligible by our claims algorithm, but the medical records revealed that each had an exclusion criterion. Finally, rather than requiring that the AMI code appear in the primary diagnosis position on the index claim, we included AMI codes appearing anywhere on the claim. This strategy opened our study up to more disconfirmed cases [15] but was more appropriate for identifying prevalent cases and study-eligible patients, as opposed to identifying incident events in drug-safety studies. This validation of the identification of study-eligible patients, as opposed to incident events, is the main way our study differs from most claims-based validation studies in the literature, and it represents an important contribution to currently available knowledge, because it opens a new avenue for patient recruitment into PCTs.

One limitation of this study was that only 61.7% of requested records were obtained. This level of retrieval is similar to several medical-record studies originating from claims databases [14,15]. The characteristics of the patients whose records were retrieved did not differ, on average, from the characteristics of the full sample of patients whose records were requested (see appendix table 1). Additionally, administrative hurdles, such as the target facility not considering our IRB approval sufficient, accounted for the large majority of unobtained records (see appendix Table 2). The similarity in patient characteristics between obtained and unobtained records and the administrative nature of the reasons for unobtained records suggest that the inability to obtain a larger percentage of requested records may not affect the study results.

The exclusion from our validation study of patients whose claims histories indicated CAD without AMI, PCI, or CABG could limit the generalizability of our findings. However, this exclusion does not create bias, as patients whose medical records indicated CAD only were not included in the numerator of our main PPV calculation. In the main ADAPTABLE study, aspirin dosing is randomized with respect to the number and type of eligibility criteria met, and study investigators have planned an analysis of heterogeneity of treatment effect by eligibility criteria.

There were two cases of aspirin-allergic patients who otherwise would have been study-eligible. We knew that claims data would not identify aspirin allergy, as confirmed by these two cases. This is a limitation of claims data, but the small number of aspirin allergy cases observed suggests that it was a minor issue in this study. The three cases of GI bleed found in the medical records but not the claims were perhaps of greater concern for the use of claims data, because GI bleed should be observable in diagnosis codes. However, both aspirin allergy and GI bleed are screened for safety in the ADAPTABLE online patient enrollment portal, so the concern of enrolling patients with excluded conditions is limited.

## 5 Conclusion

This investigation was a case study showing that claims data could be as effective in identifying subjects eligible for a pragmatic clinical trial as they are at identifying drug-safety surveillance events. The validation of the patient identification algorithm based on HealthCore's administrative claims data to identify potential ADAPTABLE study participants supports the use of claims data for recruitment of patients in pragmatic clinical trials.

## Funding source

This study was sponsored by the Patient-Centered Outcomes Research Institute. Contract for Advancement of PCORnet Infrastructure: Health Plan Data Project. HealthCore Inc., HealthCore-Anthem Research Network (HCARN), HP-1510-32545.

## Financial disclosure

Ezra Fishman, John Barron, Jade Dinh, Amanda Marshall, Rebecca Merkh, and Kevin Haynes are employees of HealthCore, Inc., a wholly owned subsidiary of Anthem, Inc.

## Conflicts of interest

Ezra Fishman, John Barron, Jade Dinh, Amanda Marshall, Rebecca Merkh, and Kevin Haynes are employees of HealthCore, Inc., a wholly owned subsidiary of Anthem, Inc.

## Disclaimer

The findings and conclusions in this report are those of the authors and do not necessarily represent the official position of Anthem or HealthCore.

## Figures and Tables

**Appendix Table 1:** Comparison of sampled patients with records obtained and abstracted vs. patients whose records were unable to be obtained/abstracted.

Characteristic	Patients whose records were not obtained by vendor	Patients whose records were obtained	P-Value for difference
n	115	185	
Age (mean, SD)	71.3 (12.7)	74.1 (11.3)	0.096
Age>65y (n, %)	87 (75.7)	150 (81.1)	0.262
Female (n, %)	43 (37.4)	70 (37.8)	0.938
Census regions (n, %)			
Northeast	15 (13.0)	31 (16.8)	0.385
South	39 (33.9)	58 (31.4)	0.645
Midwest	29 (25.2)	60 (32.4)	0.183
West	32 (27.8)	36 (19.5)	0.092
Unknown	0 (0)	0 (0)	–
Inclusion criteria (n, %):			
History of AMI	84 (73.0)	146 (78.9)	0.242
History of PCI	67 (58.3)	113 (61.1)	0.628
History of CABG	36 (31.3)	73 (39.5)	0.153
History of CAD	109 (94.8)	176 (95.1)	0.892
Enrichment factors (n, %):			
Presence of diabetes	55 (47.8)	93 (50.3)	0.681
Presence of cerebrovascular disease	53 (46.1)	96 (51.9)	0.328
Presence of peripheral artery disease	34 (29.6)	75 (40.5)	0.055
Left ventricular ejection fraction < 50%	17 (14.8)	39 (21.1)	0.173
Creatinine > 1.5 mg/dL	1 (0.9)	2 (1.1)	0.858
systolic or diastolic heart failure	44 (38.3)	87 (47.0)	0.137
LDL cholesterol > 130 mg/dL	3 (2.6)	8 (4.3)	0.442

SD = standard deviation; AMI = acute myocardial infarction; PCI = percutaneous coronary intervention; CABG = coronary artery bypass graft; CAD = coronary artery disease; LDL = low-density lipoprotein.

**Appendix Table 2:** Top reasons for unobtained medical records.

Reason	N
Provider Requires Patient Consent/IRB Approval (i.e. facility did not consider our study IRB approval sufficient)	57
Provider Refuses to Participate	31
Contacted Office Multiple Times with No Response	14
Patient not found	11

## Appendix A Supplementary data

Data Profile Data Profile

AppendixAbstractionForm AppendixAbstractionForm

## Footnotes

Appendix A Supplementary data to this article can be found online at https://doi.org/10.1016/j.conctc.2018.11.001.
