# Supplementary material for: Validation of a claims-based algorithm identifying eligible study subjects in the ADAPTABLE pragmatic clinical trial
Source: Contemp Clin Trials Commun. 2018 Nov 10;12:154–60. doi: 10.1016/j.conctc.2018.11.001 (PMC6240793; doi:10.1016/j.conctc.2018.11.001)
Supplement: AppendixAbstractionForm [file mmc2.pdf]

Abstraction Form

Please note: This is a sample highlighting the information to be included. HealthCore/Sponsor may make minimal alterations to the content and formatting prior to distribution, if necessary.

ADAPTABLE phenotype validation study

SECTION 1: PATIENT IDENTIFICATION & RECORD INFORMATION

Patient ID Code: ☐☐☐☐☐☐☐☐☐☐☐☐☐☐☐☐☐☐☐☐☐☐☐☐☐☐☐☐☐
Pre-populated patient ID code from the patient/provide list

Abstractor's Initials: ☐☐☐
Initials of abstractor

Abstraction Date (MM/DD/YYYY): ☐☐ / ☐☐ / ☐☐☐☐
Date medical record is abstracted

Gender (Claims): ☐ Male ☐ Female
Pre-populated gender from patient/provider list

Date of Birth (Claims): ☐☐ / ☐☐ / ☐☐☐☐
Pre-populated DOB from patient/provider list

Index Date (Claims): ☐☐ / ☐☐ / ☐☐☐☐
Pre-populated index date/date of interest from patient/provider list.

Chart Request Start Date (Claims): ☐☐ / ☐☐ / ☐☐☐☐
Pre-populated medical record request start date from patient/provider list
Index Date – 30 days

Chart Request End Date (Claims): ☐☐ / ☐☐ / ☐☐☐☐
Pre-populated medical record request end date from the patient/provider list
Index Date + 30 days

Chart Start Date (Chart):
Date of earliest information in medical record (MM/DD/YYYY)
☐☐ / ☐☐ / ☐☐☐☐

Chart End Date (Chart):
Date of latest information in medical record (MM/DD/YYYY)
☐☐ / ☐☐ / ☐☐☐☐

Medical Record Type:
Medical record type (choose one)
☐ Paper
☐ Electronic health record (EHR)
☐ Hybrid
If EHR or hybrid, name of system used: ____________________

Gender (Chart): ☐ Male ☐ Female ☐ Not Documented
Gender from medical record (choose one)

Date of Birth (Chart): ☐☐ / ☐☐ / ☐☐☐☐
DOB from medical record, MM/DD/YYYY

SECTION 2: MEDICAL RECORD ELIGIBILITY
Timeframe: Chart Request Start Date to Chart Request End Date
Place of service (select all that apply):
- ❑ *Hospital (inpatient)*
- ❑ *Emergency Department*
- ❑ *None of the above (IF NONE OF THE ABOVE, **END ABSTRACTION**)*

Which of the following are included in the record (select all that apply):
- ❑ *Hospital discharge summary*
- ❑ *Hospital admission summary with history*
- ❑ *Cardiologist consultation notes*
- ❑ *Emergency department discharge summary*
- ❑ *None of the above (IF NONE OF THE ABOVE, **END ABSTRACTION**)*

SECTION 3: EVIDENCE OF ATHEROSCLEROTIC CARDIOVASCULAR DISEASE (ASCVD)
Timeframe: Chart Request Start Date to Chart Request End Date

1. Does the patient have a history of myocardial infarction (MI)? *(See code list below)*
   YES | NO | NOT DOCUMENTED
   - If Yes, please provide the first page number in the chart where the information was found: ____

2. Does the patient have a history of a coronary artery bypass grafting (CABG)? *(See code list below)*
   YES | NO | NOT DOCUMENTED
   - If Yes, please provide the first page number in the chart where the information was found: ____

3. Does the patient have a history of percutaneous coronary intervention (PCI)? *(See code list below)*
   YES | NO | NOT DOCUMENTED
   - If Yes, please provide the first page number in the chart where the information was found: ____

SECTION 4: EVIDENCE OF EXCLUSION FROM ADAPTABLE CLINICAL TRIAL
Time frame: Chart Start Date to Chart End Date

4. Is there documentation of a history of allergy to aspirin?
   YES | NO
   - If Yes, please provide the first page number in the chart where the information was found: ____

5. Is there documentation of a history of significant gastrointestinal bleeding? *(See code list below)*
   YES | NO

- If Yes, please provide the first page number in the chart where the information was found: ____

6. Is there documentation of any bleeding disorder that precludes the use of aspirin? *(See code list below)*
   YES | NO
   - If Yes, please provide the first page number in the chart where the information was found: ____

Appendix: Relevant diagnosis and procedure codes

Codes for myocardial infarction:
ICD-9 diagnosis codes
'41000', '41001', '41002', '41010', '41011', '41012', '41020', '41021', '41022', '41030', '41031', '41032', '41040', '41041', '41042', '41050', '41051', '41052', '41060', '41061', '41062', '41070', '41071', '41072', '41080', '41081', '41082', '41090', '41091', '41092', '412'
-or-
ICD-10 diagnosis codes
'I2101', 'I2102', 'I2109', 'I2111', 'I2119', 'I2121', 'I2129', 'I213', 'I214', 'I220', 'I221', 'I222', 'I228', 'I229', 'I252'

Codes for CABG:
ICD-9 diagnosis code 'V4581'
or
ICD-10 diagnosis code 'Z951'
or
ICD-9 procedure codes '3610', '3611', '3612', '3613', '3614', '3615', '3616', '3617', '3619'
or
ICD-10 procedure codes '0210093', '0210098', '0210099', '021009C', '021009F', '021009W', '02100A3', '02100A8', '02100A9', '02100AC', '02100AF', '02100AW', '02100J3', '02100J8', '02100J9', '02100JC', '02100JF', '02100JW', '02100K3', '02100K8', '02100K9', '02100KC', '02100KF', '02100KW', '02100Z3', '02100Z8', '02100Z9', '02100ZC', '02100ZF', '0210344', '02103D4', '0210444', '0210493', '0210498', '0210499', '021049C', '021049F', '021049W', '02104A3', '02104A8', '02104A9', '02104AC', '02104AF', '02104AW', '02104D4', '02104J3', '02104J8', '02104J9', '02104JC', '02104JF', '02104JW', '02104K3', '02104K8', '02104K9', '02104KC', '02104KF', '02104KW', '02104Z3', '02104Z8', '02104Z9', '02104ZC', '02104ZF', '0211093', '0211098', '0211099', '021109C', '021109F', '021109W', '02110A3', '02110A8', '02110A9', '02110AC', '02110AF', '02110AW', '02110J3', '02110J8', '02110J9', '02110JC', '02110JF', '02110JW', '02110K3', '02110K8', '02110K9', '02110KC', '02110KF', '02110KW', '02110Z3', '02110Z8', '02110Z9', '02110ZC', '02110ZF', '0211344', '02113D4', '0211444', '0211493', '0211498', '0211499', '021149C', '021149F', '021149W', '02114A3', '02114A8', '02114A9', '02114AC', '02114AF', '02114AW', '02114D4', '02114J3', '02114J8', '02114J9', '02114JC', '02114JF', '02114JW', '02114K3', '02114K8', '02114K9', '02114KC', '02114KF', '02114KW', '02114Z3', '02114Z8', '02114Z9', '02114ZC', '02114ZF', '0212093', '0212098', '0212099', '021209C', '021209F', '021209W', '02120A3', '02120A8', '02120A9', '02120AC', '02120AF', '02120AW', '02120J3', '02120J8', '02120J9', '02120JC', '02120JF', '02120JW', '02120K3', '02120K8', '02120K9', '02120KC', '02120KF', '02120KW', '02120Z3', '02120Z8', '02120Z9', '02120ZC', '02120ZF', '0212344', '02123D4', '0212444', '0212493', '0212498', '0212499', '021249C', '021249F', '021249W', '02124A3', '02124A8', '02124A9', '02124AC', '02124AF', '02124AW', '02124D4', '02124J3', '02124J8', '02124J9', '02124JC', '02124JF', '02124JW', '02124K3', '02124K8', '02124K9', '02124KC', '02124KF', '02124KW', '02124Z3', '02124Z8', '02124Z9', '02124ZC', '02124ZF', '0213093', '0213098', '0213099', '021309C', '021309F', '021309W', '02130A3', '02130A8', '02130A9', '02130AC', '02130AF', '02130AW', '02130J3', '02130J8', '02130J9', '02130JC', '02130JF', '02130JW', '02130K3', '02130K8', '02130K9', '02130KC', '02130KF', '02130KW', '02130Z3', '02130Z8', '02130Z9', '02130ZC', '02130ZF', '0213344', '02133D4', '0213444', '0213493', '0213498', '0213499', '021349C', '021349F', '021349W', '02134A3', '02134A8', '02134A9', '02134AC', '02134AF', '02134AW', '02134D4', '02134J3', '02134J8', '02134J9', '02134JC', '02134JF',

'02134JW', '02134K3', '02134K8', '02134K9', '02134KC', '02134KF', '02134KW', '02134Z3', '02134Z8', '02134Z9', '02134ZC', '02134ZF'
or
CPT/HCPCS codes '33510', '33511', '33512', '33513', '33514', '33516', '33517', '33518', '33519', '33521', '33522', '33523', '33533', '33534', '33535', '33536'

Codes for PCI:
ICD-9 Diagnosis code V4582
or
ICD-10 Diagnosis code 'Z955','Z9861'
or
ICD-9 Procedure code '0066', '1755', '3601', '3602', '3605', '3606', '3607', '3609'
or
ICD-10 Procedure codes '0270046', '027004Z', '02700D6', '02700DZ', '02700T6', '02700TZ', '02700Z6', '02700ZZ', '0270346', '027034Z', '02703D6', '02703DZ', '02703T6', '02703TZ', '02703Z6', '02703ZZ', '0270446', '027044Z', '02704D6', '02704DZ', '02704T6', '02704TZ', '02704Z6', '02704ZZ', '0271046', '027104Z', '02710D6', '02710DZ', '02710T6', '02710TZ', '02710Z6', '02710ZZ', '0271346', '027134Z', '02713D6', '02713DZ', '02713T6', '02713TZ', '02713Z6', '02713ZZ', '0271446', '027144Z', '02714D6', '02714DZ', '02714T6', '02714TZ', '02714Z6', '02714ZZ', '0272046', '027204Z', '02720D6', '02720DZ', '02720T6', '02720TZ', '02720Z6', '02720ZZ', '0272346', '027234Z', '02723D6', '02723DZ', '02723T6', '02723TZ', '02723Z6', '02723ZZ', '0272446', '027244Z', '02724D6', '02724DZ', '02724T6', '02724TZ', '02724Z6', '02724ZZ', '0273046', '027304Z', '02730D6', '02730DZ', '02730T6', '02730TZ', '02730Z6', '02730ZZ', '0273346', '027334Z', '02733D6', '02733DZ', '02733T6', '02733TZ', '02733Z6', '02733ZZ', '0273446', '027344Z', '02734D6', '02734DZ', '02734T6', '02734TZ', '02734Z6', '02734ZZ', '02C03ZZ', '02C04ZZ', '02C13ZZ', '02C14ZZ', '02C23ZZ', '02C24ZZ', '02C33ZZ', '02C34ZZ', 'X2C0361', 'X2C1361'
or
CPT/HCPCS codes '92920', '92921', '92924', '92925', '92928', '92929', '92933', '92934', '92937', '92938', '92941', '92943', '92944', '92980', '92981', '92982', '92984', '92995', '92996', 'C9600', 'C9601', 'C9602', 'C9603', 'C9604', 'C9605', 'C9606', 'C9607', 'C9608', 'G0290', 'G0291'

History of significant GI bleed
ICD-9 diagnosis codes
'53100', '53101', '53120', '53121', '53140', '53141', '53160', '53161', '53200', '53201', '53220', '53221', '53240', '53241', '53260', '53261', '53300', '53301', '53320', '53321', '53340', '53341', '53360', '53361', '53400', '53401', '53420', '53421', '53440', '53441', '53460', '53461', '5781', '5307', '4560', '45620', '53082', '5693', '5780', '5789'
-or-
ICD-10 diagnosis codes
'I8501', 'I8511', 'K226', 'K228', 'K250', 'K252', 'K254', 'K256', 'K260', 'K262', 'K264', 'K266', 'K270', 'K272', 'K274', 'K276', 'K280', 'K282', 'K284', 'K286', 'K625', 'K920', 'K921', 'K922'

Bleeding disorder that precludes the use of aspirin
ICD-9 diagnosis code
'2860', '2861', '2862', '2863', '2864', '28652', '28653', '28659', '2866', '2867', '2869'
-or-
ICD-10 diagnosis code
'D65', 'D66', 'D67', 'D680', 'D681', 'D682', 'D68311', 'D68312', 'D68318', 'D6832', 'D684', 'D688', 'D689'
